# Risk-Neutrality of RND and Option Pricing within an Entropy Framework

**DOI:** 10.3390/e22080836

**Published:** 2020-07-30

**Authors:** Xisheng Yu

**Affiliations:** School of Economic Mathematics, Southwestern University of Finance and Economics, Chengdu 610074, China; yuxisheng@swufe.edu.cn

**Keywords:** risk-neutral moment, risk-neutral distribution, entropy valuation, risk-neutrality, option pricing

## Abstract

This article constructs an entropy pricing framework by incorporating a set of informative risk-neutral moments (RNMs) extracted from the market-available options as constraints. Within the RNM-constrained entropic framework, a unique distribution close enough to the correct one is obtained, and its risk-neutrality is deeply verified based on simulations. Using this resultant risk-neutral distribution (RND), a sample of risk-neutral paths of the underlying price is generated and ultimately the European option’s prices are computed. The pricing performance and analysis in simulations demonstrate that this proposed valuation is comparable to the benchmarks and can produce fairly accurate prices for options.

## 1. Introduction

The key issue in applying the risk-neutral pricing method to option pricing is to find a suitable risk-neutral pricing measure (or risk-neutral distribution, RND). With the absence of arbitrage, a perfect and complete market ensures the existence of a unique equivalent martingale measure (as the RND) [1,2]. In the realistic market system, however, many candidates are allowed for the RND due to the incompleteness, and one has to choose one particular measure by using nonparametric or model-independent methods. Some retrospective studies on nonparametric valuations can be found in [3,4,5,6].

Among those above, an entropy-based pricing approach (canonical valuation), was initialized by Stutzer [7] to European option pricing. The idea is that between two distributions (One is an empirical distribution of the underlying return, another one is its risk-neutral counterpart utilized to value the option) satisfying the martingale restriction, the one that is more “uncertain” should be selected. In general, applying the entropy criterion delivers convincing results. For instance, on the unit interval, if no constraints are given, the density with the maximum entropy then is the uniform density. Using the maximum entropy principle, the canonical approach transforms the empirical distribution of the underlying return into a risk-neutral distribution (RND, also called maximum entropy distribution or canonical martingale measure), which is then used to price option. With the martingale constraint, the resultant RND derived within the canonical valuation framework is a martingale measure and is reasonably regarded as the “best” RND for option pricing. When deriving the risk-neutral measure, the canonical approach requires no normative assumptions for the underlying dynamics but rather relies upon the available cross-sectional market-information, and for this reason, this valuation approach is nonparametric and called model-free.

Owing to the appealing feature, this entropy-based canonical valuation has been surveyed in the literature. Buchen and Kelly [8] examine the ability of canonical method to price European options in an incomplete market by extracting the RND from option prices and further discuss a relative entropy principle for option pricing in a simulated market. Shortly after, Stutzer [9] mathematically showed that, with a sole martingale constraint, the RND (canonical martingale measure) derived from his canonical framework is equivalent to the Black–Scholes measure. To shrink the feasible set of canonical measures more tightly around the correct martingale measure, Alcock and Auerswald [10] use more price-sensitive information as a second constraint by setting a specific call option to be correctly priced, to produce a more accurate option price than Stutzer’s original canonical price. Neri and Schneider [11] investigate the proposition of the density of Buchen–Kelly within the family of maximum entropy densities and find that the densities converge to that of Buchen–Kelly in the sense of relative entropy. Some recent research related to entropy-valuation can also be found in [12,13] and more recently, in [14,15].

Despite the attractiveness of *canonical valuation* and the entropy-based extensions above, they could be improved when adding more option-implied informative constraints into the corresponding entropy frame, rather than using the sole martingale condition or imposing a second constraint. Indeed, option prices contain much efficient information (e.g., the volatility smile and tail behavior etc.) about market participants’ perceptions of the underlying return which accurately captures the shape of the correct RND (see, e.g., [16,17,18,19,20]). From the perspective of statistics, for instance, a normal distribution can be identified via the first- and second-order moments. Hence, if one can retrieve the risk-neutral moments (RNMs) using option data, and incorporate the RNMs as a set of constraints into the entropy framework, then the appropriate RND can be accurately estimate from this framework so as to produce a fairly accurate price for the being-priced option.

Motivated by this, this paper sets up an entropy valuation framework by incorporating the informative RNMs serving as constraints, nesting the sole martingale constraint into Stutzer’s canonical valuation, within which the “best” risk-neutral distribution (RND) is derived as the equivalent martingale measure for pricing European options. To deeply assess the efficacy of this RNM-based entropy valuation, the accuracy of the exacted RNMs and the risk-neutralities of resultant RND are verified, and ultimately the pricing performance is fully evaluated. This proposed entropy valuation has the following advantages. First, the implementation of this method is relatively tractable due to that the method does not impose pre-assumptions on either the market structure or the underlying dynamics. Meanwhile, extracting the RNMs is also model-free. Further, the RNM constraints we used in the valuation framework nest the single martingale constraint in the original canonical valuation. Second, the informative RNMs serving as constraints can be estimated with a high accuracy by utilizing a small amount of option data, and in this way, we can learn much more about the shape of the correct RND since the option-contained information, such as volatility smile, skewness and excess kurtosis, is exploited by the RNMs. Consequently, such obtained RND can be reasonably chosen as the “best” one for option pricing, and produces a fairly precise price, as expected.

The implementation of our RNM-based entropy pricing framework proceeds in three steps. First, the RNMs are recovered using market-available option prices and incorporated, as constraints, into the entropy framework, then the RND is derived as the pricing measure within the framework. Ultimately the price of option is calculated according to the risk-neutral pricing principle. Following these, we contribute to the literature step by step: First, simulation experiments are conducted to check whether the RNMs can be accurately calculated using option prices. More importantly, the risk-neutrality of resultant RND is also experimentally verified. Finally, in the simulation setting, we present proof that the values of RNMs indeed correspond to the true values, and as expected, the risk-neutrality of the resultant risk-neutral measure (RND) is also fully confirmed. We further demonstrate the pricing efficacy of the proposed method by benchmarking the estimated price against the true price, as well as by comparing with the canonical valuation. Fortunately, all the results of those mentioned above encourage us to argue that the RNM-constrained entropy valuation framework we established offers an attractive choice for option pricing.

The remainder of this paper is constructed as follows. The RNM-based entropy valuation framework incorporating the informative RNMs is established and the pricing scheme is derived in Section 2. We present propositions of RNMs and RND and conduct simulation experiments to verify the correctness of estimated RNMs and the risk-neutrality of RND in Section 3. Section 4 provides the pricing result and analysis, and Section 5 ends with conclusions.

## 2. Entropy Valuation with RNM-Constraints

This section constructs the entropy valuation by imposing the RNM restrictions on this framework and provides the pricing scheme by deriving the RND. With the RNM constraints replacing the single martingale restriction, a more suitable RND is derived as the risk-neutral measure for option pricing. Note that, again, such obtained RND can correctly reflect the asset price behavior such as volatility smile, skewness and excess kurtosis, so that the options can be accurately priced by using this RND.

### 2.1. Pricing Scheme

We start with some notation frequently used throughout this paper. Assume the initial time *t_0_ = 0* and the underlying asset pays dividends during the life of option with a yield *q*. We denote the price of the underlying asset at time *t* by *S_t_* (0≤t≤T) and the maturity date of the option by *T*. Let the *τ*-period underlying log-return at time 𝑡 be given as the price ratio $R_{t,\tau }=\ln(S_{t+\tau }/S_{t})$, and *τ*-period 𝑗th-order RNM at time 𝑡 be defined as $m_{t,\tau }(j)=E^{\pi^{Q}}({\left[R_{t,\tau }\right]}^{j})$, where the symbol $E^{\pi^{Q}}(\cdot )$ represents the expectation operator under the risk-neutral measure $\pi^{Q}$, and *τ* can be any appropriate time period (e.g., the time to maturity or a day). A special case is where *τ* equals the time to maturity (*τ = T − t_0_*). As usual, we denote the time zero price of European option with strike *K* and maturity *T* by *C*(*T, K*) for call and *P*(*T, K*) for put.

According to Yu and Yang [20], the risk-neutral moments of gross return $m_{t,\tau }(j)(j=1,2,\ldots )$ can be expressed as an integral function of option prices as below.

**Lemma** **1** **(RNM** **Representation).** *Under the martingale pricing measure* $\pi^{Q}$ *, the risk-neutral moment (RNM)* $m_{t,\tau }(j)$ *can be retrieved from the market prices of out-of-the-money (OTM) call and put options, as follows. The* $\left(T-t_{0}\right)$ *-period first order RNM* $m_{t_{0},\tau }(1)$ *is expressed as*

(1) $$m_{t_{0},\tau }(1)=e^{\left(r-q)(T-t_{0}\right)}-e^{r(T-t_{0})}\left[\int_{S_{0}}^{\infty }\frac{1}{K^{2}}C(T,K)dK+\int_{0}^{S_{0}}\frac{1}{K^{2}}P(T,K)dK\right]-1$$

*and* $\left(T-t_{0}\right)$ *-period jth-order RNM* $m_{t_{0},\tau }(j)$ *(* j≥2 *) is given by*

(2) $$\begin{array}{l} m_{t_{0},\tau }(j)=je^{r(T-t_{0})}\times [\int_{S_{0}}^{\infty }\frac{\left(j-1)-\ln(K/S_{0}\right)}{K^{2}}{\left(\ln(K/S_{0})\right)}^{\left(j-2\right)}C(T,K)dK\\ +\int_{0}^{S_{0}}\frac{\left(j-1)-\ln(K/S_{0}\right)}{K^{2}}{\left(-\ln(K/S_{0})\right)}^{\left(j-2\right)}P(T,K)dK] \end{array}$$

*where S_0_ denotes the current underlying price, r the risk-free interest rate (continuously compounded) matching the time to the option maturity and q the dividend yield. Both r and q are annualized and supposed to be constant for a given time to maturity.*

From this lemma, RNMs are written as the integrals of option prices over a range of strike prices [*0, S_0_*] and [*S_0_, ∞*] with two singular points 0 and ∞. We discuss the calculations of RNMs later.

To construct the RNM-constrained entropy framework, we consider a sequence of historical underlying asset’s price which is used to produces the $\left(T-t_{0}\right)$-period log-return series $R_{i}=\ln(S_{t_{-(i-1)}}/S_{t_{-(i-1)}-T})$ where $t_{-i}$ is the time point prior to current time *t_0_* ( = 0) for all i=1,2,⋯,N. Suppose no foreknowledge is imposed for the asset price/return dynamics, we therefore assign each return *R_i_* equal probabilities $\pi_{i}^{P}=1/N$ as the prior empirical distribution. Hence, the return process with such measure $\pi^{P}=(\pi_{1}^{P},\cdots ,\pi_{N}^{P})$ is a sample estimate of the true asset return dynamics. Now, the RNM-constrained entropy pricing scheme faces the following problem:

(3) $$\left\{ \begin{array}{l} \pi^{Q}=\underset{\pi_{i}^{Q}>0}{\arg\min}\sum_{i=1}^{N}\pi_{i}^{Q}log(\pi_{i}^{Q}/\pi_{i}^{P})\\ s.t.\begin{array}{cc} \left\{ \begin{array}{l} \sum_{i=1}^{N}\pi_{i}^{Q}R_{i}{}^{j}=m_{t_{0},\tau }(j)\\ m_{t_{0},\tau }(0)=1 \end{array}\right. & ,j=0,1,2,\cdots \end{array},J \end{array}\right.$$

where $\pi^{Q}=(\pi_{1}^{Q},\cdots ,\pi_{N}^{Q})$ is the risk-neutral probability distribution (RND) of the underlying log-return we are seeking to price options, and as defined, *R_i_* is the log-return observations and $m_{t_{0},\tau }(j)$ is the RNMs of log-return with $t_{0}=0$ and τ=T.

### 2.2. Calculations of RNM and Derivation of RND

Within this RNM-constrained entropy model (3), the pricing scheme is then derived by specifying the calculation of RNMs and the derivation of RND.

#### 2.2.1. Calculations of RNM

Lemma 1 shows that the RNMs are the integrals of option prices over a range of strike prices on [*0, S_0_*] and [*S_0_*, *∞*] with two singular points 0 and ∞. Given a continuum of strike prices over these intervals, calculating the integrals via a numerical method is straightforward. However, only a finite number of traded options with discrete strike prices are available in a real market. Following the convention of some literature, we employ the trapezoidal method to numerically evaluate the integral and use an effective curve-fitting method to handle the option availability issue. The operational procedures are outlined as follows—for details, see [19,20].

First, the intervals of integration $\left[0,S_{0}\right)$ and $\left[S_{0},+\infty \right)$ are split into three subintervals, $\left[0,S_{0})=[0,K_{0})\cup [K_{0},K_{min})\cup [K_{min},S_{0}\right)$ and $\left[S_{0},+\infty )=[S_{0},K_{max})\cup [K_{max},K_{\infty })\cup [K_{\infty },+\infty \right)$, respectively, where $K_{min}$ is the minimum of market-available strike price and $K_{max}$ the maximum one, whereas $K_{0}$/$K_{\infty }$ is a very small/large number (in this study setting $K_{0}=0.2K_{min}$, $K_{\infty }=5K_{max}$) so that a put/call option with strike prices in $\left[0,K_{0}\right)$/$\left[K_{\infty },\infty \right)$ is valueless. The integrals over $\left[0,K_{0}\right)$ and $\left[K_{\infty },\infty \right)$ in (1) and (2) are therefore zero. Second, the integrals over other intervals need to be calculated. As mentioned above, the integrals require strike prices beyond the range of the available data, hence we need to infer the option prices corresponding to such strike prices from the given option prices. A curve-fitting method is then adopted to treat with this restriction by constructing a set of implied volatilities from observed option prices via Black–Scholes option formula (First, implied volatilities are calculated via the Black–Scholes (B–S) formula based on the selected set of option prices. Second, a cubic spline function is used to interpolate the implied volatilities and infer the implied volatilities at strike points located in [*S_0_,* *K_max_*] or [*K_min_,* *S_0_*] from the fitted function. Third, we use the Black–Scholes formula again to inversely map the inferred volatilities so as to obtain values such as the required option prices. Note that the B–S formula here is merely used as a tool to build a smooth nonlinear relation between volatility and option prices.) Third, for the integrations over $\left[K_{min},S_{0}\right)$ and $\left[S_{0},K_{max}\right)$, we use two constants (i.e., endpoint implied volatilities) to extrapolate the option prices for two intervals beyond the available range. The extrapolation is truncated at the strike points, denoted as *K_0_* and *K_∞_*. Fourth, two types of Riemann integral sums are employed when numerically approximating the integrals. Specifically, Riemann sums of the left endpoints, as well as the right endpoints, are first calculated, and their average is then used as an approximation of the required integral. In this study, we adopt a trapezoidal numerical method and each of the intervals is divided into a number (𝑚 = 80) of equidistant subintervals.

#### 2.2.2. Derivation of RND

**Theorem** **1** **(RND** **Solution).** *Assume the prior distribution is uniform* $\pi_{i}^{P}=1/N$ *, and consider a time interval* τ=T *. Then the optimal solution of equivalent martingale measure RND is obtained by solving the optimization problem (3),*

(4) $${\hat{\pi }}_{i}^{Q}=\frac{exp(\sum_{j=1}^{J}\lambda_{j}^{*}R_{i}^{j})}{\sum_{i=1}^{N}exp(\sum_{j=1}^{J}\lambda_{j}^{*}R_{i}^{j})}$$

*where the Lagrange vector* $\lambda^{*}=(\lambda_{1}^{*},\lambda_{2}^{*},\cdots \lambda_{J}^{*})$ *is found numerically by the following optimization,*

(5) $$\lambda^{*}=\underset{\lambda_{1},\cdots ,\lambda_{J}}{arg}min\sum_{i=1}^{N}exp\left(\sum_{j=1}^{J}\lambda_{j}[R_{i}^{j}-m_{t_{0},T}(j)]\right)$$

**Proof.**  Here we provide a fairly simple and easily-understood way to finish this proof, although it can also be solved by employing a dual method, see Ben–Tal [21] (pp. 264–269).

Note that Problem (3) is a strictly convex optimization and has a unique global optimal solution. Then, we employ the Lagrange multiplier method for solution seeking. The Lagrangian function for the constrained optimization problem (3) is obtained by (note that $\pi_{i}^{P}=1/N$),

$$L(\pi^{Q};\lambda_{0},\lambda )=\sum_{i=1}^{N}\pi_{i}^{Q}log(\pi_{i}^{Q})+\lambda_{0}(1-\sum_{i=1}^{N}\pi_{i}^{Q})+\sum_{j=1}^{J}\lambda_{j}[m_{t_{0},T}(j)-\sum_{i=1}^{N}\pi_{i}^{Q}R_{i}^{j}],$$

where $\lambda =(\lambda_{1},\cdots ,\lambda_{J})$ is the Lagrange multiplier. Then the first-order conditions are

$$\frac{\partial L}{\partial \pi_{i}^{Q}}=1+log(\pi_{i}^{Q})-\lambda_{0}-\sum_{j=1}^{J}\lambda_{j}R_{i}^{j}=0,$$

which leads to

(6) $$\pi_{i}^{Q}=exp[\sum_{j=1}^{J}\lambda_{j}R_{i}^{j}-(1-\lambda_{0})]$$

Summing these probabilities to one (i.e., the constraint $m_{t_{0},\tau }(0)=1$) implies

(7) $$\sum_{i=1}^{N}exp(\sum_{j=1}^{J}\lambda_{j}R_{i}^{j})=exp(1-\lambda_{0})$$

and substituting (7) into (6) yields the solution of $\pi_{i}^{Q}$, as desired,

(8) $$\pi_{i}^{Q}=\frac{exp(\sum_{j=1}^{J}\lambda_{j}R_{i}^{j})}{\sum_{i=1}^{N}exp(\sum_{j=1}^{J}\lambda_{j}R_{i}^{j})}$$

Applying formula (8) to constraint equations $\sum_{i=1}^{N}\pi_{i}^{Q}R_{i}{}^{j}=m_{t_{0},T}(j)$ produces,

$$\sum_{i=1}^{N}\frac{exp(\sum_{j=1}^{J}\lambda_{j}R_{i}^{j})R_{i}^{j}}{\sum_{i=1}^{N}exp(\sum_{j=1}^{J}\lambda_{j}R_{i}^{j})}=m_{t_{0},T}(j)$$

equivalent to the following by rearranging,

$$\sum_{i=1}^{N}exp\left(\sum_{j=1}^{J}\lambda_{j}[R_{i}^{j}-m_{t_{0},T}(j)]\right)\left(R_{i}^{j}-m_{t_{0},T}(j)\right)\left(exp[\sum_{j=1}^{J}\lambda_{j}m_{t_{0},T}(j)]\right)=0$$

$$\mathrm{or},\;\sum_{i=1}^{N}exp\left(\sum_{j=1}^{J}\lambda_{j}[R_{i}^{j}-m_{t_{0},T}(j)]\right)\left(R_{i}^{j}-m_{t_{0},T}(j)\right)=0$$

in which the left-hand side is exactly a partial derivative,

(9) $$\sum_{i=1}^{N}exp(\sum_{j=1}^{J}\lambda_{j}R_{i}^{j})=exp(1-\lambda_{0})$$

Now returning to optimization problem (5), the derived Equation (9) is the first condition of problem (5), and note that the objective function in (5) is strictly convex, therefore the Lagrange multiplier *λ* in (8) satisfying (9) must be the unique solution to problem (5). □

### 2.3. Risk-Neutral underlying Paths and Option Price

The resultant distribution ${\hat{\pi }}^{Q}=({\hat{\pi }}_{1}^{Q},\cdots ,{\hat{\pi }}_{N}^{Q})$ in (4) represents the occurrence of the empirical log-return $R_{i}=\ln(S_{t_{-(i-1)}}/S_{t_{-(i-1)}-T})$ and will serve as the risk-neutral probability measure we are seeking for option pricing. Now with the risk-neutral probability ${\hat{\pi }}^{Q}$, an independent random sample of log-returns, $\tilde{R}=({\tilde{R}}_{1},\cdots ,{\tilde{R}}_{M})$, can be drawn from the set of historical log-returns ${\left\{R_{i}\right\}}_{i=1}^{N}$ by employing an inverse transform method ([22] (pp. 230–232)), and then utilized to directly generate *M* risk-neutral price paths for the underlying asset at option maturity *T*,

$$S_{T}^{\left(k\right)}=S_{0}{\tilde{R}}_{k}(k=1,2,\cdots ,M),$$

where ${\tilde{R}}_{k}$ is the *k*-th sample of the log-return corresponding to the *k*-th price path, and *S*_0_ is the underlying price at initial time *t*_0_ = 0.

Next, we calculate the European option price. Since the simulated sample of underlying price paths is under the risk-neutral measure ${\hat{\pi }}^{Q}$, as specified above, it is therefore risk-neutral. Hence, according to the risk-neutral pricing method, directly averaging the discounted payoff of all paths yields the final value of the option. Specifically, a call/put option maturating at time *T* with strike *K*, can be valued as the following,

(10) $$(\mathrm{for}\;\mathrm{call}\;\mathrm{option})\;\;\;C(T,K)=\frac{1}{M}\sum_{k=1}^{M}e^{-r(T-t_{0})}{\left(S_{T}^{\left(k\right)}-K\right)}^{+}$$

(11) $$(\mathrm{for}\;\mathrm{put}\;\mathrm{option})\;\;\;P(T,K)=\frac{1}{M}\sum_{k=1}^{M}e^{-r(T-t_{0})}{\left(K-S_{T}^{\left(k\right)}\right)}^{+}$$

where *r* is the interest rate matching the time *t*_0_ to *T* and $a^{+}$ takes the maximum of zero and *a*.

## 3. Verification of Correctness of Extracted RNMs and Risk-Neutrality of RND

Due to the significance of RNMs in deriving RND, it is indispensable to verify the correctness of calculated RNMs in Lemma 1, as well as the risk-neutrality of obtained RND in (4). To complete this verification, a market that provides a true value for each RNM and a real RND is needed, so that we can facilitate the comparisons between the estimated RNMs and the true RNMs, and between the derived RND and the real RND. Considering such requirements, we choose a simple but effective Black–Scholes (B–S) market in which the exact RNMs and RND can be easily derived.

### 3.1. Correctness of the Estimated RNMs

Within the B–S setting, a geometric Brownian motion (GBM) is assumed for the underlying process *S_t_*,

(12) $$\frac{dS_{t}}{S_{t}}=(\mu -q)dt+\sigma d\omega_{t}$$

where *μ* is the growth rate, *q* the continuous dividend yield and $\omega_{t}$ the standard Wiener process. The log-return is then normally distributed and given by,

(13) $$R_{t_{0},T}=(\mu -q-\frac{\sigma^{2}}{2})T+\sigma \sqrt{T}\varepsilon$$

where ε is standard normal.

According to the definition of RNM of log-return, the RNM in B–S world is then expressed as $m_{t_{0},T}^{BS}(j)=E^{\pi^{BS}}({\left[R_{t_{0},T}\right]}^{j})$, where $\pi^{BS}$ is the B–S risk-neutral measure. Conceptually, within a B–S market, the RNMs $m_{t_{0},T}(j)$(reminding that $m_{t_{0},T}(j)=E^{\pi^{Q}}({\left[R_{t_{0},T}\right]}^{j})$) and the RND $\pi^{Q}$ derived from our entropy valuation framework should be the same with $m_{t_{0},T}^{BS}(j)$ and $\pi^{BS}$, respectively. Meanwhile it is notable that the B–S (risk-neutral measure) can be uniquely determined by the first two moments of log-return, $m_{t_{0},T}^{BS}(1)$ and $m_{t_{0},T}^{BS}(2)$, because the log-price is normally distributed following Equation (13) and the risk-neutral distribution is exactly characterized by its first two moments (i.e., (*μ* − *q*) and volatility *σ*). Consequently, in this context of B–S setting, we choose to use two RNM constraints in our entropy framework (3) by taking J = 2.

The following Theorem 2 states the correctness of the estimated RNMs $m_{t_{0},T}(j)$ in Lemma 1 by verifying the equivalence relation between RNMs $m_{t_{0},T}(j)$ and B–S RNM $m_{t_{0},T}^{BS}(j)$ *(j* = 1,2).

**Theorem** **2**  **(Equivalence** **of** **RNM).** *Within the B–S setting, denote the first two order moments of log-return under the B–S martingale measure by* $m_{t_{0},T}^{BS}(j)=E^{\pi^{BS}}({\left[R_{t_{0},T}\right]}^{j})$ *(j = 1,2), where* $\pi^{BS}$ *is the B–S risk-neutral measure, then for the risk-neutral moments* $m_{t_{0},T}(j)$ *using options detailed in Lemma 1, we obtain:*

(14) $$m_{t_{0},T}(1)=m_{{}_{t_{0},T}}^{BS}(1)=(r-q-\frac{\sigma^{2}}{2})(T-t_{0})$$

(15) $$m_{t_{0},T}(2)=m_{{}_{t_{0},T}}^{BS}(2)={\left[(r-q-\frac{\sigma^{2}}{2})(T-t_{0})\right]}^{2}+\sigma^{2}(T-t_{0})$$

**Proof.**  Without any loss of generality, assume t_0_ = 0 and q = 0. Then from Equations (1)–(2), the first- and second-order RNMs are simplified as, respectively,

(16) $$m_{T}(1)=e^{rT}-e^{rT}[\int_{S_{0}}^{\infty }\frac{1}{K^{2}}C(T,K)dK+\int_{0}^{S_{0}}\frac{1}{K^{2}}P(T,K)dK]-1$$

(17) $$m_{T}(2)=2e^{rT}[\int_{S_{0}}^{\infty }\frac{1-\ln(K/S_{0})}{K^{2}}C(T,K)dK+\int_{0}^{S_{0}}\frac{1-\ln(K/S_{0})}{K^{2}}P(T,K)dK]$$

**A-1.**  First, we show that $m_{T}(1)=(r-\frac{\sigma^{2}}{2})T$.

Note that in B–S world, the call price is expressed by $C(T,K)=S_{0}N(d_{1})-Ke^{-rT}N(d_{2})$, and put by $P(T,K)=-S_{0}N(-d_{1})+Ke^{-rT}N(-d_{2})$, where *N*(•) is the cumulative distribution function of standard normal distribution. All other letters have the conventional meanings in the sense of Black–Scholes setting, hence not explained here, and purely for convenience, we denote $a=(r+\sigma^{2}/2)T$, $b=\sigma \sqrt{T}$ and $c=(r-\sigma^{2}/2)T$.

By direct calculations (double integrals involved),

$$\begin{array}{l} \int_{S_{0}}^{\infty }\frac{1}{K^{2}}C(T,K)dK\\ =\frac{S_{0}}{K^{2}}\int_{S_{0}}^{\infty }N(d_{1})dK-\frac{e^{-rT}}{K}\int_{S_{0}}^{\infty }N(d_{2})dK\\ =[N(\frac{a}{b})-e^{-a+\frac{b^{2}}{2}}N(\frac{a}{b}-b)]-e^{-rT}[cN(\frac{c}{b})+\frac{b}{\sqrt{2\pi }}e^{-\frac{c^{2}}{2b^{2}}}]. \end{array}$$

Similarly,

$$\begin{array}{l} \int_{0}^{S_{0}}\frac{1}{K^{2}}P(T,K)dK\\ =-e^{-rT}[cN(-\frac{c}{b})-\frac{b}{\sqrt{2\pi }}e^{-\frac{c^{2}}{2b^{2}}}]-[e^{-a+\frac{b^{2}}{2}}N(b-\frac{a}{b})-N(-\frac{a}{b})] \end{array}$$

Summing them up and substituting the sum into (16) yield,

$$m_{T}(1)=e^{rT}-e^{rT}[1-(e^{-a+\frac{b^{2}}{2}}+ce^{-rT})]-1=(r-\frac{\sigma^{2}}{2})T.$$

**A-2.**  Second, the following is to prove $m_{T}(2)={\left[(r-\frac{\sigma^{2}}{2})T\right]}^{2}+\sigma^{2}T$.

Although two more integrals, $\int_{S_{0}}^{\infty }\frac{ln(K)}{K^{2}}C(T,K)dK$ and $\int_{0}^{S_{0}}\frac{ln(K)}{K^{2}}P(T,K)dK$ are involved, it is not difficult but merely a little bit complicated. Using a similar way, it follows that

$$\begin{array}{l} m_{T}(2)\\ =2e^{rT}[\int_{S_{0}}^{\infty }\frac{1-\ln(K/S_{0})}{K^{2}}C(T,K)dK+\int_{0}^{S_{0}}\frac{1-\ln(K/S_{0})}{K^{2}}P(T,K)dK]\\ =2e^{rT}[\int_{S_{0}}^{\infty }\frac{1}{K^{2}}C(T,K)dK+\int_{0}^{S_{0}}\frac{1}{K^{2}}P(T,K)dK]-2e^{rT}[\int_{S_{0}}^{\infty }\frac{\ln(K/S_{0})}{K^{2}}C(T,K)dK+\int_{0}^{S_{0}}\frac{\ln(K/S_{0})}{K^{2}}P(T,K)dK]\\ =(2e^{rT}-2c-2)+2e^{rT}[\int_{S_{0}}^{\infty }\frac{\ln(S_{0}/K)}{K^{2}}(S_{0}N(d_{1})-Ke^{-rT}N(d_{2}))dK+\int_{0}^{S_{0}}\frac{\ln(S_{0}/K)}{K^{2}}(-S_{0}N(-d_{1})+Ke^{-rT}N(-d_{2}))dK]\\ =2e^{rT}-2c-2+2e^{rT}[e^{-rT}\frac{b^{2}}{2}+e^{-rT}\frac{c^{2}}{2}-1+e^{-\frac{2a-b^{2}}{2}}(1+a-b^{2})]\\ ={\left[(r-\frac{1}{2}\sigma^{2})T\right]}^{2}+\sigma^{2}T \end{array}$$

as desired.

**B.**  *Now* return to the RNMs under B–S measure, $m_{{}_{T}}^{BS}(1)=(r-\frac{\sigma^{2}}{2})T$ and $m_{{}_{T}}^{BS}(2)={\left[(r-\frac{\sigma^{2}}{2})T\right]}^{2}+\sigma^{2}T$.

Recall that, $m_{T}^{BS}(j)=E^{\pi^{BS}}({\left[R_{T}\right]}^{j})$ *(j = 1,2)* by definition, and $R_{T}=(\mu -\frac{\sigma^{2}}{2})T+\sigma \omega_{T}$ by formula (13).

Applying the Girsanov theorem ([23] (p. 212)), $\omega_{t}^{BS}:=\omega_{t}-\frac{r-\mu }{\sigma }t$ (0 ≤ *t* ≤ *T*) is the standard Brownian motion under the B–S measure, then it is immediate that

$$\begin{array}{l} m_{{}_{T}}^{BS}(1)=E^{\pi^{BS}}[(\mu -\frac{\sigma^{2}}{2})T+\sigma \omega_{T}]\\ =(\mu -\frac{1}{2}\sigma^{2})T+\sigma E^{\pi^{BS}}(\omega_{T}^{BS}+\frac{r-\mu }{\sigma }T),\\ =(r-\frac{1}{2}\sigma^{2})T \end{array}$$

and

$$\begin{array}{l} m_{{}_{T}}^{BS}(2)=E^{\pi^{BS}}({\left[(\mu -\frac{\sigma^{2}}{2})T+\sigma \omega_{T}\right]}^{2})\\ ={\left(\mu -\frac{1}{2}\sigma^{2}\right)}^{2}T^{2}+[2\sigma (\mu -\frac{1}{2}\sigma^{2})T^{2}]E^{\pi^{BS}}(\omega_{T}^{BS}+\frac{r-\mu }{\sigma }T)+\sigma^{2}E^{\pi^{BS}}[{\left(\omega_{T}^{BS}+\frac{r-\mu }{\sigma }T\right)}^{2}]\\ ={\left[(r-\frac{1}{2}\sigma^{2}){}^{T}\right]}^{2}+\sigma^{2}T \end{array}$$

Thus, the proof is completed. □

Clearly, from Theorem 2, the extracted RNMs from option prices through (1) and (2) are the same as the true RNMs under the B–S setting. Hence, the RNMs are exactly risk-neutral with which the derived RND in (4) is consequently a risk-neutral martingale measure.

In addition, we further conduct simulations to check the accuracy of RNM estimates via formulae (1)–(2) following the procedures specified in Section 2.2.1, so that the correctness of obtained RNMs can be confirmed by comparing them with the corresponding true RNMs.

Given the initial time *t_0_* = 0, expiration *T* = 1, interest rate *r* = 0.05, dividend yield *q* = 0.02, and volatility *σ* = 0.2, the B–S RNMs $m_{t_{0},T}^{BS}(j)$ (*j* = 1, 2) can be easily calculated using (14)–(15). Meanwhile, according to Section 2.2.1, for an underlying asset’s price $S_{0}$, numerically computing RNMs via integral expressions (1)–(2) requires several pairs of “market-traded” options *C(T, K)* and *P(T, K)*. Within the B-S world, we then generate a set of OTM call and put options as the “market-available” options. In the simulations, for fully checking the accuracy of estimating RNM, we consider five levels of underlying price *S_0_* = 48, 50, 52, 54, 56 and for each level, four pairs (We numerically calculate the RNMs according to integral expressions (1)–(2) by using various numbers of OTM options, the unreported results find no significant difference in the resultant estimates of RNM. Consider the accuracy of estimates and option-availability in real marketplace, four pairs of OTM options are chosen) of OTM options with 4-point increment strikes (See Table 1 as below) are generated as the “real” market options, with which the RNMs $m_{t_{0},T}(j)$(*j* = 1, 2) are calculated.

Now, with four pairs of OTM calls and puts for each underlying price above as, for instance, (34, 50), (38, 54), (42, 58) and (46, 62) for 48, we can estimate RNMs following the procedures in Section 2.2.1 via the trapezoidal rule integration method by setting $K_{\infty }=5K_{\max}$, $K_{0}=0.2K_{min}$ and the number of nonoverlapping subintervals *m =* 80. Taking the integral $\int_{S_{0}}^{K_{\max}}\frac{1}{K^{2}}C(T,K)dK$ as an example, it can be accurately approximated as

$$\int_{S_{0}}^{K_{\max}}\frac{1}{K^{2}}C(T,K)dK\approx \frac{1}{2}[\sum_{i=1}^{m}(\frac{1}{K_{i-1}{}^{2}}C(T,K_{i-1})+\frac{1}{K_{i}{}^{2}}C(T,K_{i}))\Delta K],$$

where $\Delta K=(K_{\max}-S_{0})/m$ , $K_{i}=S_{0}+i\Delta K$ for i∈[0,m] and $C(T,K_{i})$ is obtained via interpolation using four available call prices. The estimates of RNM from (1)–(2) and the real values of RNM are shown in Table 2 for five underlying prices.

As can be seen from Table 2, the RNM estimates are the same as the real (theoretical) values (retaining four digits after the decimal point). This demonstrates that four pairs of options can effectively capture the shape of the underlying distribution due to the accurate moment estimates. *Furthermore*, the estimated values of each RNM are almost indistinguishable over a range of underlying prices. This indicates that, as expected, two moments by (1)–(2) are exactly “risk-neutral” and have nothing to do with the growth rate *μ* and current asset price *S*_0_. Conceptually, the risk-neutral moments of log-return in B–S world are only determined by the drift term and volatility term rather than the underlying asset price, which corresponds to formulae (14)–(15).

### 3.2. Risk-Neutrality of the Derived RND

So far, the correctness of calculated RNMs $m_{t_{0},T}(j)$ is already confirmed, and subsequently, as an illumination of concept, the resultant probability measure ${\hat{\pi }}^{Q}=({\hat{\pi }}_{1}^{Q},\cdots ,{\hat{\pi }}_{N}^{Q})$, as the risk-neutral measure for option pricing, should be exactly “risk-neutral”. To further confirm the risk-neutrality of measure ${\hat{\pi }}^{Q}$, we depict two sets of risk-neutral probabilities ${\hat{\pi }}_{i}^{Q(1)}$ and ${\hat{\pi }}_{i}^{Q(2)}$ using two different growth rates and show the indistinguishableness by comparing the resultant risk-neutral probability distributions, as well as the estimated density functions.

**Figure 1:**
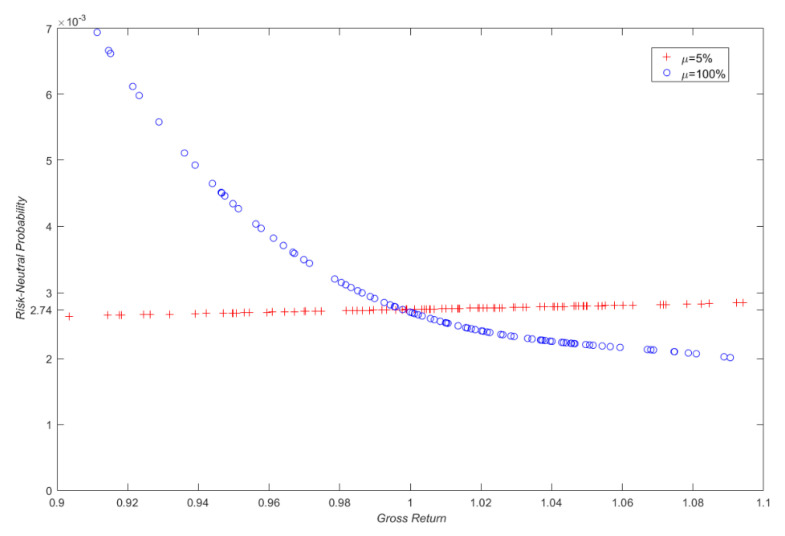
Two sets of risk-neutral probabilities based on geometric Brownian motion (GBMs) with two drift rates of 5% and 100%. Note that, for clarity, only 70 points among 365 historical gross returns are shown.

**Figure 2:**
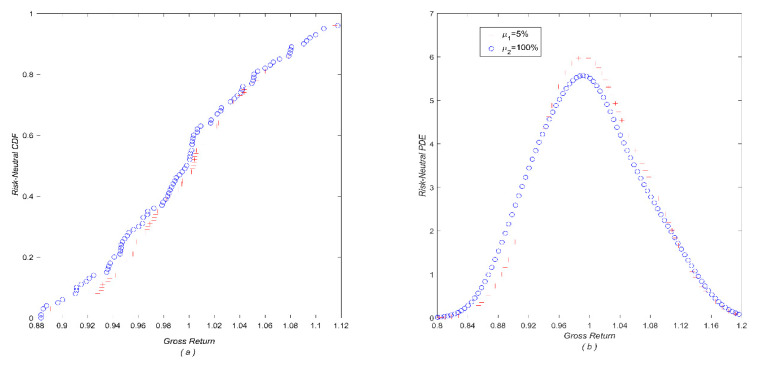
(**a**) Two risk-neutral CDFs based on GBMs with two growth rates of 5% and 100%; (**b**) the corresponding probability density estimates (PDEs). Note that only part of risk-neutral gross returns is illuminated for clarity in both figures.

First, with the parameter setting (*t_0_* = 0, *T* = 1, *r* = 0.05, *q* = 0.02, *σ* = 0.2) in Section 3.1 and according to Equation(12), two series of 365 $\left(T-t_{0}\right)$-period gross log-returns are produced using the risk-neutral growth rate (*μ_1_* = *r* = 5%) and an unrealistic rate (*μ_1_* = 100%), and then treated as the “historical” log-returns *R_i_* (*i = 1,2,…, 356*) as defined in Section 2.1. Second, with these two samples of historical log-returns, two corresponding RNDs for both cases are calculated as ${\hat{\pi }}_{i}^{Q(1)}=({\hat{\pi }}_{1}^{Q(1)},{\hat{\pi }}_{2}^{Q(1)},\cdots ,{\hat{\pi }}_{365}^{Q(1)})$ and ${\hat{\pi }}_{i}^{Q(2)}=({\hat{\pi }}_{1}^{Q(2)},{\hat{\pi }}_{2}^{Q(2)},\cdots ,{\hat{\pi }}_{365}^{Q(2)})$ via Equation (4), respectively. Finally, we plot two sets of estimated risk-neutral probabilities ${\hat{\pi }}_{i}^{Q(1)}$ and ${\hat{\pi }}_{i}^{Q(2)}$ in Figure 1, and the corresponding risk-neutral cumulative distribution functions (CDFs) and probability density estimates (PDEs) are shown in Figure 2.

Figure 1 depicts the resultant risk-neutral probabilities ${\hat{\pi }}_{i}^{Q(1)}$ with a growth rate of 5% and ${\hat{\pi }}_{i}^{Q(2)}$ with 100% respectively, and two major findings can be found as expected. First, as shown in Figure 1, all the risk-neutral probabilities in the case of 5% growth rate, are roughly equal to 0.00274 ( = 1/365). This result is quite explicable since the inputs (gross returns) in this case are produced in the risk-neutral world (growth rate set to be interest rate *μ_1_ = r = 5%*). In contrast, the probability curve decreases with gross return when the growth rate is at 100%. This is also understandable since the lower returns require higher probabilities so as to offer a risk-neutral measure. Second, although the curves of the probabilities for 5% and 100% growth rates look quite different, their corresponding CDFs/ PDEs are nearly indistinguishable (Figure 2). This is because two CDFs/PDFs must be very similar in order to yield approximately the same risk-neutral result. This result exactly implies the risk-neutrality of the probabilities.

## 4. Pricing Performance and Analysis

With the verifications of risk-neutrality RND, it is conceptually reasonable to claim that our proposed entropy-based scheme can provide an appropriate RND as the risk-neutral pricing measure and consequently a quite high pricing accuracy is ensured. This section processes a further evaluation of the proposed method by conducting simulation tests using two different drift rates *μ* in a Black–Scholes (B–S) environment, as well as in a more realistic stochastic volatility model of Heston [24]. In addition, our proposed method is also compared with the canonical valuation with a single martingale constraint. Note that it is adequate to concentrate on the pricing of European call options for the following reasons: First, given the call price, the put price is immediate due to put–call parity. More importantly, formulae (10)–(11) indicate that the same risk-neutral price paths are used between pricing call and put options, which would also result in an accurate price for the put option as the call is accurately priced.

### 4.1. Performance in a B–S Environment

In a B–S market, the GBM for underlying price *S_t_* is provided by Equation (12) and the log-return $R_{t_{0},T}$ is calculated as (13). To make a *more comprehensive analysis*, various levels of moneyness (*S_0_/ K* for call) and time to maturity are considered, and we assign a reasonable set of parameter values as follows.

- Valuation date: *t*_0_ = 0
- Expiration date (in year): *T* = 1/12, 1/4, 1/2, 3/4, 1
- Strike price *K* = 52
- Initial asset price: *S*_0_ = 4 8, 50, 52, 54, 56
- Risk-free interest rate: *r* = 5%
- Drift rate: *μ*_1_ = 5%, *μ*_2_ = 100%
- Volatility: *σ* = 20%
- Dividend yield (Without any loss of generality but merely a computational convenience, here we set dividend yield q = 0.): *q* = 0

To enable the calculations of RNMs $m_{t_{0},T}(1)$ and $m_{t_{0},T}(2)$, as specified previously, four pairs of OTM options (with different strikes) are generated as the “real” market options for each underlying price *S*_0_. In this B–S setting, we use the strike prices in Table 2, e.g., four calls with strikes (34, 38, 42, 46) and four puts with (50, 54, 58, 62) to produce four pairs of call and put options as B–S market options to estimate the RNMs for each price of *S*_0_ with a time to maturity *T*.

For each time to maturity *T*, 365 log-returns are drawn from (12), then following the operational steps detailed in Section 3.2, two corresponding RNDs with different drift rate are derived and used as the risk-neutral measures for pricing options.

Table 3 reports the results with two different growth rates using the proposed RNM-based entropy method (RNM–Entropy) and canonical valuation (As previously mentioned, canonical valuation utilizes the sole martingale constraint, $\sum \pi_{i}^{Q}e^{R_{i}}=e^{r(T-t_{0})}$, and the canonical price for option can be calculated by using the same procedure as that of our RNM-constrained entropy method.) (Canonical). Each reported price is the average of resultant values based on five independent simulations, in each of which 5000 risk-neutral underlying price paths were generated for computing the call price by using formula (10).

First, as is shown in Table 3, the resultant prices from our entropy method are rather close to the “true” values (B–S prices) for both drift rates over a range of underlying prices, and those price estimates from the canonical method are also approximately the true values. It is noteworthy that, as aforementioned, in the Black–Scholes world, the canonical valuation (with a single martingale constraint) is mathematically shown to offer a price equal to the B–S price. This is the reason why the canonical estimates are also close to the B–S prices in this simulation. Meanwhile, our RNM-constrained entropy approach still outperforms the canonical valuation by using the pricing errors. Second, the differences in absolute value between the estimates and the B–S prices are all below 0.16% in the case of a growth rate of 100%, with the largest being 0.1574% for the option with an asset price of 48 and a short term of 1/12, whereas the difference is up to 1.3674% for canonical valuation. Third, in the case of a risk-neutral growth rate of 5%, all are below 0.08% with the largest only 0.0787% for the RNM–entropy method and the largest is 1.2589% for the canonical method. It is quite understandable for both the highest differences, considering that in this situation the option is out-of-the-money and the true value (B–S price) is very small at 0.1271 which could result in a “big” error when computing the percentage difference.

Furthermore, for each price estimate of the RNM–entropy method in both growth rates of 5% and 100%, the pricing error is so small that the difference between two corresponding estimates is slight. This finding suggests again that this proposed method is independent of the drift rate and the resultant RND is consequently risk-neutral, as previously outlined in Figure 1 and Figure 2, which ensures high pricing precision. Consequently, it appears that the proposed RNM-based entropy valuation is completely comparable to the benchmarking Black–Scholes formula for European options.

### 4.2. Performance in a Stochastic Volatility Model

In order to conduct a more realistic test of proposed entropy method, following the convention of much literature (e.g., [25]), we investigate the performance of our method, as well as the canonical valuation, using the stochastic volatility (SV) model of Heston [24], where the asset price is assumed to obey

(18) $$\frac{dS_{t}}{S_{t}}=\mu dt+\sqrt{\nu_{t}}d\omega_{s,t}$$

and the return’s variance follows an Ornstein–Uhlenbeck process

(19) $$d\nu_{t}=\kappa (\theta -\nu_{t})dt+\eta \sqrt{\nu_{t}}d\omega_{v,t}$$

where, as usual, *κ* is the speed of mean reversion, *θ* the long-run variance, *η* the volatility of the volatility generating process, and $d\omega_{s,t}$ and $d\omega_{v,t}$ are Wiener processes with correlation *ρ*.

The appealing feature of this setup is that this model retains an integral-involved closed form solution for the European option price. To bypass the substantial bias (see [26]), we adopt the Gauss–Kronrod quadrature method (Kahaner et al. [27]) rather than the commonly-known Euler discretization when calculating the integrals, then the option price under this SV model is computed.

In the same manner as Haley and Walker [25], the same parameter values for SV model (18)–(19) are provided as seen below,

- Drift rate: *μ* = 10%
- Mean reversion: κ = 3
- Long-run mean: θ = 4%
- Volatility: *η* = 40%
- Correlation: ρ = −0.5,

and the factors of the option being valued are as follows,

- Valuation date: *t*_0_ = 0
- Expiration date (in year): *T* = 1/12, 1/4, 1/2, 3/4, 1
- Strike price: *K* = 52
- Risk-free interest rate: *r* = 5%
- Dividend yield: *q* = 0

According to the procedures in Section 3.2 and following the same computational details as Section 4.1, a sample of log-return is generated using the parameter values above and the RNMs can be calculated so that the risk-neutral paths are simulated, and ultimately the option prices are computed using the RNM–entropy method and canonical valuation respectively. The pricing results are outlined in Table 4 for both methods. Each of the prices resulted from our entropy scheme (RNM–entropy) and canonical method (Canonical) is the averaged values over five independent simulations and each simulation generates 5000 risk-neutral sample price paths.

Again, the comparisons facilitated in Heston’s SV model exploit the superb pricing power of the proposed RNM-constrained entropy scheme. First, observations from Table 4 show that the estimated price from RNM–entropy scheme is fairly close to the “true” value (Heston price) for each combination of asset price (or moneyness) and time to maturity, and this finding in the Heston model is in line with that in the B–S world. Second, using the deviation judging measure–difference indicator, the pricing error is definitely acceptable since the largest absolute difference value is merely 0.0611% for the RNM–entropy scheme in this more realistic circumstance. Contrarily, for the canonical valuation, the magnitude of pricing error, by the difference indicator, is relatively large compared with that resulting from the RNM–entropy valuation. This is because, in this stochastic volatility environment, the efficient information on volatility’s dynamics is readily expressed by the risk-neutral moments (RNMs) which are incorporated into our entropy pricing scheme, while the canonical valuation cannot correctly capture the volatility’s behavior since it fails to use more constraints, like these RNM constraints, except the martingale restriction. More importantly, the slight pricing error using a difference indicator reveals that the RNMs can also effectively capture the features of risk-neutral distribution such as the volatility within Heston’s SV model. It should be noted that, as in the B–S world, there is no discernible relation between the pricing accuracy and moneyness (*S_0_ / K*) or time to maturity for both methods.

In brief, these pricing results described in Table 3 and Table 4 indicate that European calls (hence put) can be priced rather accurately by our RNM–entropy approach in both simulated markets, regardless of an ideal environment or a more realistic model. It is noteworthy that, by comparison with the classical canonical valuation, the extracted informative RNMs from the option “market” play a significant role for the RNM–entropy approach to create the superb pricing performance, as RNMs can capture the shape of RND accurately enough.

## 5. Conclusions

This article establishes a risk-neutral moment-constrained (RNM-constrained) entropic pricing framework, within which the optimal RND (an equivalent martingale measure) is achieved via the maximum entropy principle, as the “best” risk-neutral pricing measure to produce rather accurate prices for options.

The informative RNMs can be retrieved from a set of market-available options and utilized to correctly capture the features of the RND (such as the volatility, skewness, and kurtosis, etc.) for option pricing. We provide the general expression for extracting the RNMs and prove that the calculated RNMs using the expressions are the same as the true values of RNM in a Black–Scholes setting. Further, the risk-neutrality of such obtained RND within the RNM-based entropy frame is deeply verified in the simulation experiments by showing the independence of RND on the underlying growth rate.

The pricing performance of our entropy pricing method is fully evaluated in simulation environments including a more realistic stochastic volatility (SV) scenario as well as the Black–Scholes market. The simulation tests, in a Black–Scholes (B–S) world, demonstrate that the resultant prices with both different drift rates from the entropy method are very close to the true values (B–S prices), and that the pricing error for each option is too slight. Hence, this RNM–entropy valuation sounds comparable to the right benchmark B–S formula in the B–S setting. Within the Heston’s SV model, this entropy method, again, prices options fairly well for a range of combinations including moneyness and time to maturity. The results under this SV model reveal that the pricing bias for each combination is so small that the difference between the price from our entropy scheme and that from the SV model has no obvious discernible pattern with moneyness or maturity. It should also be noted that, through the comparisons between RNM–entropy method and canonical valuation facilitated in both markets, the imposed RNM restrictions in our RNM–entropy framework are of great importance since the RNMs contain a lot of useful market information such as volatility smile, skewness and excess kurtosis, which can be effectively reflected into the derived risk-neutral measure.

In summary, this proposed RNM-constrained entropy valuation is conceptually and practically appealing since it does not impose any underlying structural assumption but relies more on the effective information included in the marketplace, and in this way, the resultant price of the option can match the market behavior and be close enough to the actual market price of the option. Therefore, in principle, this entropy method can be applied in any other artificial environment and actual markets due to its ability to achieve a martingale measure close enough to the correct one. Hence, it is not unreasonable to imagine that this proposed RNM-based entropy method provides an attractive and effective way for option pricing.

## Figures and Tables

**Table 1:** Strike prices of generated “market-observed” out-of-the-money (OTM) options in a Black–Scholes (B–S) world for a range of initial underlying prices.

Underling Price *S_0_*	48	50	52	54	56
Strikes of OTM Calls	34, 38, 42, 46	36, 40, 44, 48	38, 42, 46, 50	40, 44, 48, 52	42, 46, 50, 54
Strikes of OTM Puts	50, 54, 58, 62	52, 56, 60, 64	54, 60, 64, 68	56, 58, 62, 66	58, 62, 64, 70

**Table 2:** Comparisons between estimated risk-neutral moment (RNM) and real value in a B–S world for a range of initial underlying prices.

Underling price *S_0_*	48	50	52	54	56
1st-order RNM	Real value: 0.0100				
	0.0100	0.0100	0.0100	0.0100	0.0100
2nd-order RNM	Real value: 0.0401				
	0.0401	0.0401	0.0401	0.0401	0.0401

*Notes*: The first two order moment estimates for log-return with various underlying’s prices are compared to the theoretical values in the Black–Scholes market with parameters *r* = 0.05, *q* = 0.02, *σ* = 0.2 and *T* = 1. These moments are recovered by using only 4 pairs of options. For both moments, cells in the bottom row represent estimated values while the top is the real (theoretical) value.

**Table 3:** Averaged price estimates of calls across a range of asset prices (with *K* = 52) in B–S world with two growth rates.

Asset Price *S*_0_	Time to Maturity (year)	*μ*_1_ = 5%				B–S Prices(True Values)	*μ*_2_ = 100%			
		RNM–Entropy		Canonical			RNM–Entropy		Canonical	
		Estimates	Diff (%)	Estimates	Diff (%)		Estimates	Diff (%)	Estimates	Diff (%)
48	1/12	0.1272	0.0787	0.1287	1.2589	0.1271	0.1273	0.1574	0.1288	1.3674
	1/4	0.7392	0.0406	0.7407	0.2436	0.7389	0.7395	0.0812	0.7423	0.4628
	1/2	1.6319	−0.0123	1.6317	−0.0245	1.6321	1.6317	−0.0245	1.6314	−0.0417
	3/4	2.4430	0.0082	2.4424	−0.0164	2.4428	2.4437	0.0368	2.4422	−0.0246
	1	3.1920	−0.0438	3.1928	−0.0188	3.1934	3.1918	−0.0501	3.1915	−0.0595
50	1/12	0.4899	0.0408	0.4913	0.3267	0.4897	0.4902	0.1021	0.4918	0.4312
	1/4	1.4161	0.0424	1.4148	−0.0495	1.4155	1.4163	0.0565	1.4146	−0.0653
	1/2	2.4961	0.0441	2.4934	−0.0641	2.4950	2.4964	0.0561	2.4930	−0.0788
	3/4	3.4095	−0.0352	3.4118	0.0323	3.4107	3.4092	−0.0440	3.4122	0.0426
	1	4.2345	−0.0165	4.2345	−0.0165	4.2352	4.2351	−0.0024	4.2343	−0.0203
52	1/12	1.3067	0.0306	1.3053	−0.0766	1.3063	1.3071	0.0612	1.3050	−0.1011
	1/4	2.4003	0.0208	2.3994	−0.0167	2.3998	2.4005	0.0292	2.3993	−0.0220
	1/2	3.5816	−0.0140	3.5805	−0.0447	3.5821	3.581	−0.0307	3.5800	−0.0590
	3/4	4.5610	−0.0132	4.5601	−0.0329	4.5616	4.5608	−0.0175	4.5598	−0.0405
	1	5.4331	−0.0221	5.4361	0.0331	5.4343	5.4312	−0.0570	5.4367	0.0437
54	1/12	2.6331	−0.0190	2.6354	0.0683	2.6336	2.6328	−0.0304	2.6360	0.0902
	1/4	3.6821	−0.0244	3.6833	0.0081	3.6830	3.6825	−0.0136	3.6834	0.0107
	1/2	4.8774	−0.0266	4.8769	−0.0369	4.8787	4.8772	−0.0307	4.8763	−0.0487
	3/4	5.8785	−0.0459	5.8773	−0.0663	5.8812	5.8784	−0.0476	5.8761	−0.0862
	1	6.7745	−0.0443	6.7749	−0.0384	6.7775	6.7741	−0.0502	6.7733	−0.0614
56	1/12	4.3407	−0.0253	4.3431	0.0299	4.3418	4.3410	−0.0184	4.3434	0.0359
	1/4	5.2165	−0.0364	5.2165	−0.0364	5.2184	5.2163	−0.0402	5.2156	−0.0546
	1/2	6.3563	−0.0236	6.3560	−0.0283	6.3578	6.3564	−0.0220	6.3556	−0.0340
	3/4	7.3468	−0.0367	7.3462	−0.0449	7.3495	7.3465	−0.0408	7.3456	−0.0530
	1	8.2449	−0.0412	8.2509	0.0315	8.2483	8.2445	−0.0461	8.2514	0.0378

*Notes:* The values in “B–S prices” column are computed using Black–Scholes formula as the “true” prices. “Estimates” columns report the price estimates with the risk-neutral growth rates of 5% (i.e., *μ*_1_ = *r*) and 100%, from our RNM-constrained entropy method and the canonical approach. “Diff” columns measure the difference between price estimate and “true” price (B–S price), which is calculated by dividing the estimated price minus the “true” price by the “true” price, that is, $(p^{estimate}-p^{true})\times 100/p^{true}$. For the reported price estimates, each of them is the averaged value over five independent simulations and each of the simulations generates 5000 sample price paths.

**Table 4:** Averaged price estimates of calls across a range of asset prices (with *K* = 52) in Heston’s stochastic volatility (SV) model.

Asset Price *S*_0_	Time to Maturity(Year)	Heston (True Prices)	RNM–Entropy		Canonical	
			Estimates	Difference (%)	Estimates	Difference (%)
48	1/12	1.1963	1.1970	0.0611	1.2044	0.6732
	1/4	2.7061	2.7075	0.0503	2.7150	0.3287
	1/2	3.9327	3.9319	−0.0215	3.9309	−0.0465
	3/4	4.7408	4.7427	0.0405	4.7387	−0.0454
	1	5.3936	5.3906	−0.0552	5.3904	−0.0602
50	1/12	1.9506	1.9517	0.0547	1.9586	0.4102
	1/4	3.6527	3.6553	0.0699	3.6491	−0.0990
	1/2	4.9873	4.9903	0.0609	4.9795	−0.1559
	3/4	5.8583	5.8558	−0.0434	5.8639	0.0957
	1	6.5574	6.5562	−0.0185	6.5539	−0.0541
52	1/12	2.9381	2.939	0.0302	2.9329	−0.1758
	1/4	4.7514	4.7527	0.0271	4.7495	−0.0405
	1/2	6.1631	6.1621	−0.0163	6.1550	−0.1310
	3/4	7.0835	7.0846	0.0155	7.0761	−0.1039
	1	7.8203	7.8184	−0.0249	7.8291	0.1131
54	1/12	4.1476	4.1467	−0.0211	4.1567	0.2200
	1/4	5.9904	5.9888	−0.0275	5.9919	0.0251
	1/2	7.4492	7.4467	−0.0329	7.4404	−0.1182
	3/4	8.4063	8.4021	−0.0496	8.3870	−0.2296
	1	9.1727	9.1772	0.0492	9.1598	−0.1410
56	1/12	5.5532	5.5517	−0.0278	5.5596	0.1157
	1/4	7.3555	7.3524	−0.0423	7.3458	−0.1318
	1/2	8.8344	8.8366	0.0249	8.8253	−0.1035
	3/4	9.8164	9.8123	−0.0416	9.7998	−0.1693
	1	10.6052	10.6102	0.0471	10.6182	0.1224

*Notes:* The values in the “Heston” column are obtained by assuming a Heston’s SV model, and naturally regarded as the “true” prices. The “Estimates” columns report the price estimates from our entropy scheme and canonical approach. The “Difference” columns measure the difference between price estimate and “true” price, which is calculated by dividing the estimated price minus the “true” price by the “true” price, that is, $(p^{estimate}-p^{true})\times 100/p^{true}$. For the reported price estimates, each of them is the averaged values over five independent simulations and each of the simulations generates 5000 sample price paths.
